# Implementation and performance of the South African Triage Scale at Kenyatta National Hospital in Nairobi, Kenya

**DOI:** 10.1186/s12245-019-0221-3

**Published:** 2019-02-11

**Authors:** Ali A. Wangara, Katherine M. Hunold, Sarah Leeper, Frederick Ndiawo, Judith Mweu, Shaun Harty, Rachael Fuchs, Ian B. K. Martin, Karen Ekernas, Stephen J. Dunlop, Michèle Twomey, Alice W. Maingi, Justin Guy Myers

**Affiliations:** 1 Accident and Emergency Department, Kenyatta National Hospital, PO Box 3956-00200, Nairobi, Kenya; 2 Department of Emergency Medicine, The Ohio State University, Columbus, OH USA; 3 Department of Emergency Medicine, University of Maryland Prince George’s Hospital Center, Maryland, MD USA; 4 Critical Care Unit, Kenyatta National Hospital, Nairobi, Kenya; 5 Department of Emergency Medicine, The University of Cincinnati, Cincinnati, OH USA; 6 Department of Biostatistics, FHI 360 & UNC Gillings School of Global Public Health, Chapel Hill, NC USA; 7 Department of Emergency Medicine, Medical College of Wisconsin, Milwaukee, WI USA; 8 Department of Emergency Medicine, St. Joseph Hospital, Denver, CO USA; 9 Department of Emergency Medicine, Hennepin County Medical Center, Minneapolis, MN USA; 10 University Hospital, Bern, Switzerland; 11 Department of Emergency Medicine, University of North Carolina at Chapel Hill School of Medicine, 170 Manning Drive, CB 7594, Chapel Hill, NC 27599 USA

**Keywords:** Accident and emergency medicine, Triage, East Africa

## Abstract

**Introduction:**

Triage protocols standardize and improve patient care in accident and emergency departments (A&Es). Kenyatta National Hospital (KNH), the largest public tertiary hospital in East Africa, is resource-limited and was without A&E-specific triage protocols.

**Objectives:**

We sought to standardize patient triage through implementation of the South African Triage Scale (SATS). We aimed to (1) assess the reliability of triage decisions among A&E healthcare workers following an educational intervention and (2) analyze the validity of the SATS in KNH’s A&E.

**Methods:**

Part 1 was a prospective, before and after trial utilizing an educational intervention and assessing triage reliability using previously validated vignettes administered to 166 healthcare workers. Part 2 was a triage chart review wherein we assessed the validity of the SATS in predicting patient disposition outcomes by inclusion of 2420 charts through retrospective, systematic sampling.

**Results:**

Healthcare workers agreed with an expert defined triage standard for 64% of triage scenarios following an educational intervention, and had a 97% agreement allowing for a one-level discrepancy in the SATS score. There was “good” inter-rater agreement based on an intraclass correlation coefficient and quadratic weighted kappa. We analyzed 1209 pre-SATS and 1211 post-SATS patient charts and found a non-significant difference in undertriage and statistically significant decrease in overtriage rates between the pre- and post-SATS cohorts (undertriage 3.8 and 7.8%, respectively, *p* = 0.2; overtriage 70.9 and 62.3%, respectively, *p* < 0.05). The SATS had a sensitivity of 92.2% and specificity of 37.7% for predicting admission, death, or discharge in the A&E.

**Conclusion:**

Healthcare worker triage decisions using the SATS were more consistent with expert opinion following an educational intervention. The SATS also performed well in predicting outcomes with high sensitivity and satisfactory levels of both undertriage and overtriage, confirming the SATS as a contextually appropriate triage system at a major East African A&E.

**Electronic supplementary material:**

The online version of this article (10.1186/s12245-019-0221-3) contains supplementary material, which is available to authorized users.

## Strengths

- Kenyatta National Hospital is the largest tertiary hospital in East Africa and this is the first study to assess the performance of the South African Triage Scale (SATS) in this setting
- We used previously validated paper-based vignettes to train and test reliability among the majority of health care workers in the accident and emergency department
- We conducted a sensitivity and specificity analysis to benchmark our results against future studies
- Our implementation committee utilized the best available evidence to inform our operational intervention and evaluate the SATS effectiveness in the A&E

## Limitations

- Paper-based triage vignettes are inherently limited compared to live patients but represent the best proxy for triage studies
- Our retrospective chart review limits our understanding of the depth to which triage providers used the SATS flowchart to designate patient triage levels
- Current benchmarks for triage standards and formulas vary widely, limiting our comparisons to recent published literature
- We did not assess resource utilization or length of stay in relation to the SATS

## Background

Triage is a foundation in the development of modern emergency care [1]. Triage practices are specialized based on the available resources, social situations, and pre-defined triage criteria.

In the developing world, triage is underutilized and is often an ineffective area of the health system [2]. Limited triage training, “gestalt” decision making, and lack of formal standardized triage systems result in inconsistent triage assignments [3] which can jeopardize patients with emergent medical conditions. Improving triage in resource-limited settings has demonstrated acceptable reliability and validity of various triage systems [4–8], and a possible reduction in pediatric mortality [9–11].

However, accident and emergency (A&E) triage scales designed for high-income countries report widely varying degrees of validity, reliability, and outcomes [12]. These variations make it difficult to predict which triage system is “ideal” for a particular context, especially for low- and middle-income countries (LMICs) [13]; therefore, dedicated studies in these environments are required. The South African Triage Scale (SATS), which was developed in resource-limited settings of South Africa [14], has demonstrated good reliability and validity in a number of studies of similar low-resourced settings [4, 5, 8, 15–18].

In Kenya, emergency care and trauma systems are considered “underdeveloped” [19] and there is no nationally accepted A&E triage system [20, 21]. Similarly, Kenyatta National Hospital (KNH), the region’s largest referral hospital, lacked standardized triage procedures in the A&E. The prior triage practice classified patients into three levels based on triage nurse clinical gestalt: Red, Yellow, and Green (emergency, urgent, and non-urgent, respectively). Patients whom were taken directly to the resuscitation room bypassed triage and were not assigned a category. A triage committee formed in November 2014 aimed to address this shortcoming and formally adopted the SATS of the South African Triage Group [22].

### Objectives

This study aimed to evaluate the success of implementation of the South African Triage Scale in KNH by (1) assessing the reliability of triage decisions by triage providers following an educational intervention and (2) analyzing the validity of the SATS at KNH’s A&E, comparing prior triage practice with the newly implemented triage protocol. Further, this project serves to address one of the four foundational challenges of acute care in sub-Saharan Africa, as outlined by consensus from the African Federation for Emergency Medicine (AFEM), which is that “healthcare facilities often lack an integrated approach to triage, resuscitation, and stabilization of acutely ill patients.” [23] To our knowledge, there is no published literature on the implementation of the SATS in Kenya or any public, tertiary A&E department with this high patient volume.

## Methods

### Setting

KNH is an 1800-bed tertiary care facility and the largest public hospital in East Africa. Emergency services at KNH are provided by the A&E and the pediatric emergency unit, which evaluates children 12 and under. Injured children and all patients over age 12 are directed to the A&E. The 2014 patient census of these combined areas was 120,249, with 69,294 patients treated in the A&E [24]. Full-time and part-time medical officers and nurses, as well as nursing and emergency medical technician (EMT) students, staff the A&E. Many nurses have completed an additional 1-year emergency nursing certification course, a nursing training program unique to KNH.

### Triage committee

The triage committee was formed in November 2014 to implement a formal triage system in hopes of improving patient outcomes, as demonstrated in previous studies [25]. The SATS has demonstrated adequate triage performance and is the most extensively studied triage scale in LMICs across Africa and into Asia [4, 5, 8, 15–17, 26, 27]. Supported by this available evidence and expert opinion, the decision was made to implement SATS at KNH. Relevant stakeholders included A&E physicians, nurses, health information officers, and administrative leaders. The system is coded by emergent, very urgent, urgent, and routine, as described elsewhere [27]. Triage acuity levels are derived from a logical flow diagram that incorporates the patient’s chief complaint, vital signs, mobility, presence of trauma, and additional investigations, such as blood glucose or pregnancy test [22]. The scale encourages the role of the senior provider or healthcare worker in “over-ruling” the flow diagram when additional clinical information warrants.

### Data analysis—triage training and reliability

Prior to implementation of SATS, all personnel involved in implementation (A&E nurses, registration officers, senior A&E leadership, A&E physicians) completed a 2-day SATS training course. The training course utilized standardized SATS training materials, and we incorporated additional simulation exercises to improve comprehension and application. All were invited to participate in post-training testing that included 25 clinical triage vignettes for calculating reliability [5]. To assess intra-rater reliability, each question was scored for agreement with experts as “exact” (i.e., having the same response as the experts), as “exact or within 1” (combining responses that were the same as experts’ or differed by one category), or were incorrect.

The intraclass coefficient (ICC) using a two-way random effects model, an established statistic in similar contexts and equivalent to a weighted kappa, was used to measure inter-rater reliability of triage decisions after training [5, 26, 28]. We also used quadratic weighted Fleiss’ kappa (QWK) to measure the inter-rater reliability and to compare with the ICC, since the QWK has been used in several prior triage studies [7, 26, 29]. Any missing test questions were handled with a distinct weight when computing the QWK. We interpreted the ICC and QWK for clinical contexts in the conventional manner, as proposed by Cicceti et al. (agreement ratings scale: < 0.4 poor, 0.4–.059 fair, 0.60–0.74 good, 0.75–1.00 excellent) [30, 31].

### Data analysis—validity

A minimal sample size of 1189 in each group was sought to detect a 5% difference using a two-sided *t*-test of proportions with level of significance of 0.05 and power of 80%, as previously reported [32]. SATS was implemented in April of 2015. We assessed the validity of the SATS in predicting patient disposition outcomes by conducting a systematic, retrospective A&E chart review by comparison of January 2015 (pre-SATS, q3 sampling) and July 2015 (post-SATS, q4 sampling) charts. These were determined to be adequate sampling intervals and time periods a priori based on known average patient volumes. We followed previously published methods for calculating overtriage, undertriage, and a sensitivity/specificity analysis [33], as suggested by Lentz et al. in order to standardize triage results among studies [34]. The pre-SATS numerator in our overtriage definition was limited to only Red patients who was discharged from the A&E, since there was no “very urgent” level in the prior triage practice.

$$ {\displaystyle \begin{array}{c}\mathrm{Undertriage}\%=\left(1-\mathrm{Sensitivity}\right)\\ {}\left(\mathrm{patients}\kern0.34em \mathrm{triaged}\kern0.34em \mathrm{high}\kern0.34em \mathrm{acuity}\kern0.2em \mathrm{who}\kern0.2em \mathrm{were}\kern0.34em \mathrm{actually}\kern0.2em \mathrm{low}\hbox{-} \mathrm{acuity}/\mathrm{all}\kern0.2em \mathrm{low}\hbox{-} \mathrm{acuity}\kern0.34em \mathrm{patients}\right)\\ {}\frac{\mathrm{Routine}\left(\mathrm{orGreen}\right)\mathrm{Admitted}+\mathrm{Routine}\left(\mathrm{orGreen}\right)\mathrm{DiedinAE}}{\mathrm{AllAdmitted}+\mathrm{AllDiedinAEpatients}}\end{array}} $$

$$ {\displaystyle \begin{array}{c}\mathrm{Overtriage}\%=\left(1\hbox{-} \mathrm{Specificity}\right)\\ {}\left(\mathrm{patients}\ \mathrm{triaged}\ \mathrm{high}\ \mathrm{acuity}\ \mathrm{who}\ \mathrm{were}\ \mathrm{actually}\ \mathrm{low}\hbox{-} \mathrm{acuity}/\mathrm{all}\ \mathrm{low}\hbox{-} \mathrm{acuity}\ \mathrm{patients}\right)\\ {}\frac{\mathrm{Emergent}\ \left(\mathrm{or}\ \mathrm{Red}\right)+\mathrm{Very}\ \mathrm{Urgent}+\mathrm{Urgent}\ \left(\mathrm{or}\ \mathrm{Yellow}\right)\ \mathrm{Discharged}}{\mathrm{All}\ \mathrm{Discharged}\ \mathrm{patients}}\end{array}} $$

Data analysis was conducted with STATA 14 (Stata Corp, College Station, TX) with the exception of the quadratic weighted analysis using Fleiss’ kappa, which was conducted with AgreeStat 2015.1 (AgreeStat 2015.1 for Excel Windows/Mac User’s Guide, Advanced Analytics, Maryland, USA).

## Results

### Triage training and reliability

There were 166 test takers that were compared. Test answers were compared to the expertly defined standard and revealed a 64% exact agreement with expert and a 97% agreement with expert within one triage category. The ICC was 0.66 (95% CI 0.54–0.79) and a chance corrected agreement correlation using Fleiss’ QWK of 0.63 (95% CI 0.49–0.76), representing a “good” agreement. When individual triage acuity categories were assessed, we found a variable percentage of correct triage answers for very urgent (72%) vs urgent (61%), routine (83%), and emergency categories (51%).

### Validity

Patients in the pre- and post-SATS cohorts were similar in age, gender, and disposition. Admission rates were also similar between cohorts (28% pre-SATS and 29% post-SATS). Nearly 1/3 of both cohorts were documented as trauma patients (Table 1).

**Table 1:** Characteristics of chart samples for validity analysis

	Pre-SATS (*n* = 1209) *n* (%)		Post-SATS (*n* = 1211) *n* (%)	
Age				
≤ 2 months	3	(0)	6	(1)
2 months–3 years	54	(4)	50	(4)
3–12 years	64	(5)	70	(6)
13–64 years	936	(77)	934	(77)
≥ 65 years	102	(8)	112	(9)
Missing	50	(4)	39	(3)
Sex				
Male	624	(52)	596	(49)
Female	584	(48)	611	(50)
Unknown/missing	1	(0)	4	(1)
Time of arrival				
7 am–3 pm	533	(44)	547	(45)
3 pm–11 pm	472	(39)	448	(37)
11 pm–7 am	203	(17)	214	(18)
Missing	1	(0)	2	(0)
Trauma				
Yes	380	(31)	349	(29)
No	454	(38)	781	(64)
Unknown/missing	375	(31)	81	(7)
Disposition				
Admit	351	(29)	335	(28)
Died in A&E	27	(2)	11	(1)
Discharged	659	(55)	740	(61)
Left without being seen	33	(3)	16	(1)
Left against medical advice	5	(0)	7	(1)
Unknown/missing	144	(12)	102	(8)

**Fig. 1:**
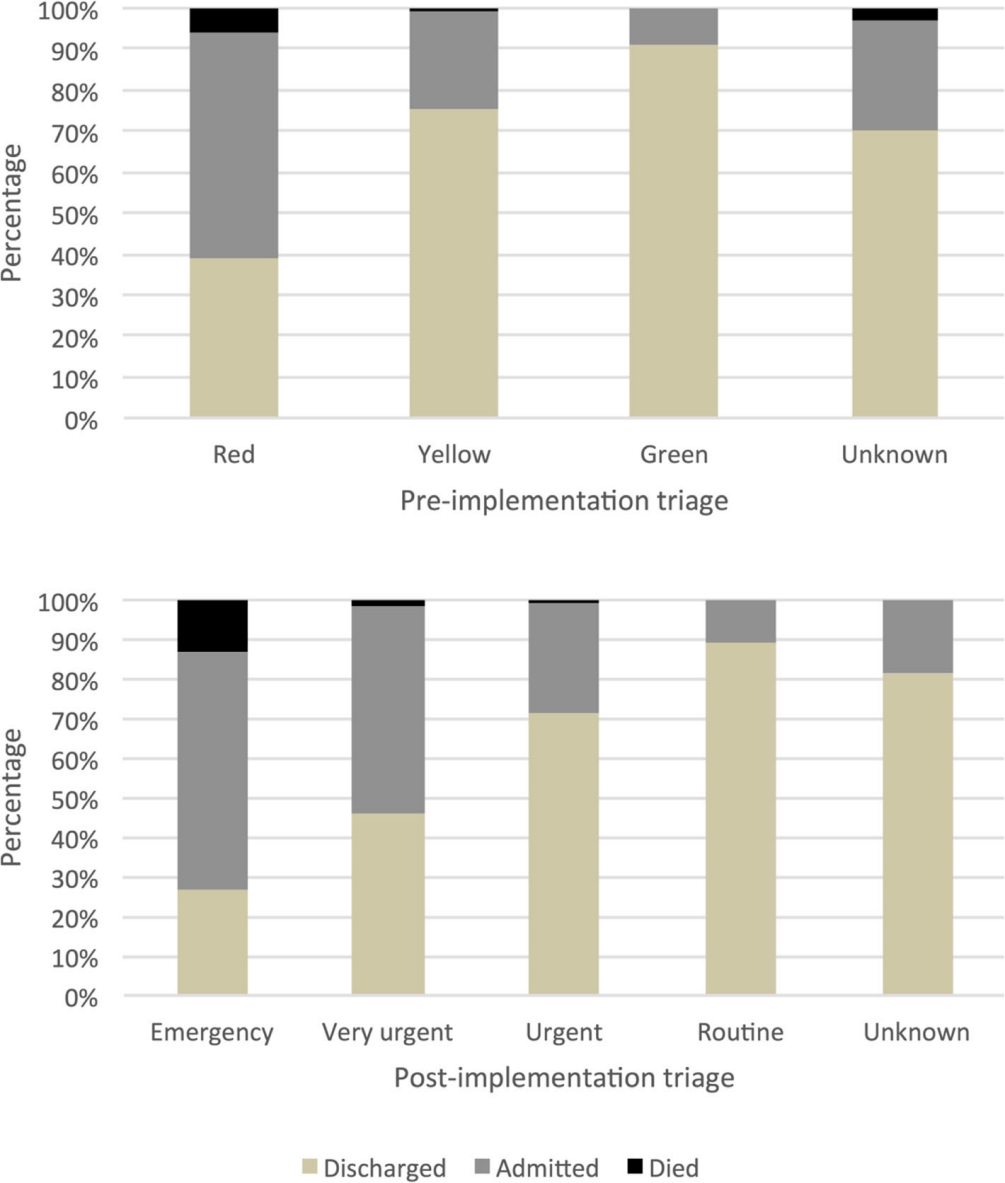
Comparison of triage patterns, by % acuity, pre-SATS and post-SATS implementation

When comparing pre- and post-SATS, there were statistically significant differences in overall undertriage and overtriage rates between some of but not all the pre- and post-SATS overall cohorts (Table 3). Figure 1 demonstrates the percentages of patients in each triage category that were admitted, discharged, or died in the A&E. Pre-SATS, 61% of Red, 24% of Yellow, and 9% of patients were admitted or died, while the remainder were discharged. Post-SATS, the pattern was similar with 73% of emergent, 54% of very urgent, 29% of urgent, and 11% of routine patients were admitted or died.

A lesser proportion of patients were actually triaged emergent post-SATS (2.4%) than pre-SATS (20.5%) (Table 2). A greater proportion of emergent patients were admitted or died in the A&E (73%), when compared to pre-SATS Red patients (61%). Conversely, a lesser proportion of emergent patients were discharged (26%), when compared to pre-SATS Red patients (39%) (Fig. 1).

**Table 2:** Triage patient designations and disposition, pre-SATS and post-SATS implementation

	Pre-SATS								Post-SATS							
	Triage category	Admitted *n* (%)		Died in A&E *n* (%)		Discharged *n* (%)		Row total	Triage category	Admitted *n* (%)		Died in A&E *n* (%)		Discharged *n* (%)		Row total
Overall	Red	211	(55)	22	(57)	151	(39)	384	Emergency	27	(60)	6	(13)	12	(27)	45
	Yellow	98	(24)	3	(1)	316	(76)	417	Very urgent	137	(53)	3	(1)	118	(46)	258
	Green	14	(9)	0	(0)	145	(91)	159	Urgent	131	(39)	2	(18)	331	(45)	464
	Unknown	18	(27)	2	(3)	47	(70)	67	Routine	27	(11)	0	(0)	222	(89)	249
	Total	341	(33)	27	(3)	659	(64)	1027	Unknown	13	(19)	0	(0)	57	(81)	70
									Total	335	(31)	11	(1)	740	(68)	1086
Pediatric (≤ 12 years)	Red	25	(53)	1	(2)	21	(45)	47	Emergency	8	(100)	0	(0)	0	(0)	8
	Yellow	15	(31)	0	(0)	34	(69)	49	Very urgent	16	(55)	0	(0)	13	(45)	29
	Green	0	(0)	0	(0)	10	(100)	10	Urgent	12	(22)	0	(0)	42	(78)	54
	Unknown	0	(0)	0	(0)	6	(100)	6	Routine	3	(19)	0	(0)	13	(81)	16
	Total	40	(36)	1	(1)	71	(63)	112	Unknown	2	(18)	0	(0)	9	(82)	11
									Total	41	(35)	0		77	(65)	118
Adult (> 12 years)	Red	172	(55)	17	(5)	124	(40)	313	Emergency	16	(47)	6	(18)	12	(35)	34
	Yellow	79	(22)	3	(1)	271	(77)	353	Very urgent	113	(52)	3	(1)	101	(47)	217
	Green	13	(9)	0	(0)	131	(91)	144	Urgent	118	(30)	2	(1)	278	(70)	398
	Unknown	18	(30)	2	(3)	40	(67)	60	Routine	24	(11)	0	(0)	202	(89)	226
	Total	282	(32)	22	(3)	566	(65)	870	Unknown	11	(19)	0	(0)	47	(81)	58
									Total	282	(30)	11	(1)	640	(69)	933

The SATS demonstrated an undertriage rate of 7.8% with a sensitivity of 92.2% and an overtriage rate of 62.3% with a specificity of 37.7%. Using this same definition, the pre-SATS undertriage rate was 3.8% with a sensitivity of 96.2% and an overtriage rate of 70.9% with a specificity of 29.1%. Our pediatric (ages 12 and under) overtriage rate was similar pre-SATS (77.5%) to post-SATS (71.4%) (*p* = 0.40) and the undertriage rate was also similar pre-SATS (0%) to post-SATS (7.7%) (*p* = 0.07) (Table 3). The positive and negative predictive values of pre-SATS (96.6 and 91.2) were similar to SATS (91.6 and 89.2) (Table 3). (see Additional file 1).

**Table 3:** Undertriage and overtriage rates during the pre- and post-SATS periods and test characteristics (of prior triage practice and SATS) at KNH. Sample sizes for each population are as defined in Table 2

	Pre-SATS	Post-SATS	*p* value
Undertriage (overall)	3.8%	7.8%	0.20
Adults (> 12 years)	4.3%	8.2%	0.06
Pediatric (≤ 12 years)	0.0%	7.3%	0.07
Overtriage (overall)	70.9%	62.3%	< 0.01
Adults (> 12 years)	69.8%	61.1%	< 0.01
Pediatric (≤ 12 years)	77.5%	71.4%	0.40
Overall			
Sensitivity	96.2	92.2	
Specificity	29.1	37.7	
Positive predictive value (admission)	95.6	91.6	
Negative predictive value (discharge)	91.2	89.2	

## Discussion

### Reliability

Our study demonstrates the successful performance of the SATS implemented at KNH. A&E providers exhibited sufficient triage knowledge and reliability using the SATS at KNH.

Our inter-rater reliability measure, the ICC, exhibited a “good” clinical agreement [30, 31]. These results match those found among nurses utilizing similar SATS validation training vignettes [26, 35].

### Overtriage/undertriage and benchmarking

Our results demonstrate a similar undertriage rate (3.8 to 7.8%, *p* = 0.2) with a statistically decreased overtriage rate (70.9 to 62.3%, *p* < 0.01). However, both prior triage practice and the SATS reveal acceptable undertriage and overtriage rates that fall within the boundaries of prior published rates [18, 33, 36] but outside the American College of Surgeons – Committee on Trauma (ACS-COT) guidelines [37], which has been used as a benchmark in some studies [4, 8]. A brief literature review of published studies analyzing SATS implementation reveals undertriage rates ranging between 0.3 and 16% and overtriage rates ranging between 4.3 and 67.8% [18, 33, 36]. These studies utilize different methods to determine under- and overtriage rates which makes comparison and benchmarking triage a moving target [13, 25, 34]. Importantly, the appropriate standard in this setting has not been established. The ACS-COT triage guidelines may not be the appropriate standard for this emergency care context as they were established for US trauma systems, which have high material and workforce resources. In addition, these are non-evidence-based guidelines proposed for pre-hospital (EMS) trauma patients, being referred to a trauma center, rather than the diverse medical and trauma case mix found in A&E departments.

### Patient stratification and overtriage

There appears to be an improved stratification of patients using a four-level system rather than the prior three-level system. The goal for triage times with SATS levels are as follows: Red, immediate; Orange, 10 min; Yellow, 1 h; and Green, 4 h. “Red” patients require immediate, focused healthcare worker attention and typically, more resources. In the pre-SATS cohort, 20.5% (211) were triaged “Red,” and in the post-SATS cohort, 2.4% (27) were triaged “emergent” patients. If the suggested time to provider standard of “immediate” for Red and emergent patients was adhered to, this represents a significant decrease in the number of patients to be seen immediately. In addition, the disposition profile of triaged emergent patients appears to be improved with SATS, since proportionally more were admitted (61 to 73%) and proportionally less discharged (39 to 26%). The overall improved specificity (from 29.2 to 37.8%) seems to supports this, although this must be interpreted cautiously since a three-level system was compared to a four-level system (Table 3). A disaster triage mantra “if everyone is immediate, then no one is immediate” applies universally to low-resource settings. In a resource-limited setting, the inappropriate allocation of resources (potentially occurring from overtriage) could be life threatening for another patient requiring those services.

### Pediatric SATS

We also assessed the performance of the SATS in the subset of pediatric patients treated in the A&E. The adult A&E evaluates pediatric burn and trauma patients while the remainder of pediatric patients are evaluated in a separate unit. Our SATS pediatric overtriage rate was 71.4% and undertriage rate was 7.3% (Table 3). In an emergency department in Botswana, the SATS pediatric overtriage rate was 28.2% and undertriage rate was 21.9%. Further, in an emergency care multicenter study of pediatrics in South Africa, the SATS had an overtriage rate of 45.5% and an undertriage rate of 9.0%. These wide variations in pediatric triage values, and as demonstrated in a recent systematic reviews of pediatric triage scales [38, 39], reflect the overall difficulty of assessing the performance and quality of triage of children in LMICs.

## Limitations

We used case scenarios (triage vignettes) for assessing inter- and intra-rater reliability following our education conference. While this represents a proxy for assessing live patients, it certainly is not the same as evaluating a sick patient in person. Assessment of live patients for triage reliability also has inherent limitations and would be difficult to assess with this quantity of health care workers. Prior research demonstrated a moderate to high level of agreement when live cases were compared to paper case scenarios; however, it is unclear which method is more accurate [40].

A lack of separate reliability analysis among physicians and nurses may be considered a limitation; however, our assessment has “real-world” applicability. Even with dedicated triage nurses at KNH, at times, other members of the medical team, including students, are called upon to perform triage. We aimed for the entire medical team to have an understanding and appreciation for this new system. Our SATS training workshop demonstrated effectiveness at producing reliable triage decisions between health care workers in the KNH A&E.

**Fig. 2:**
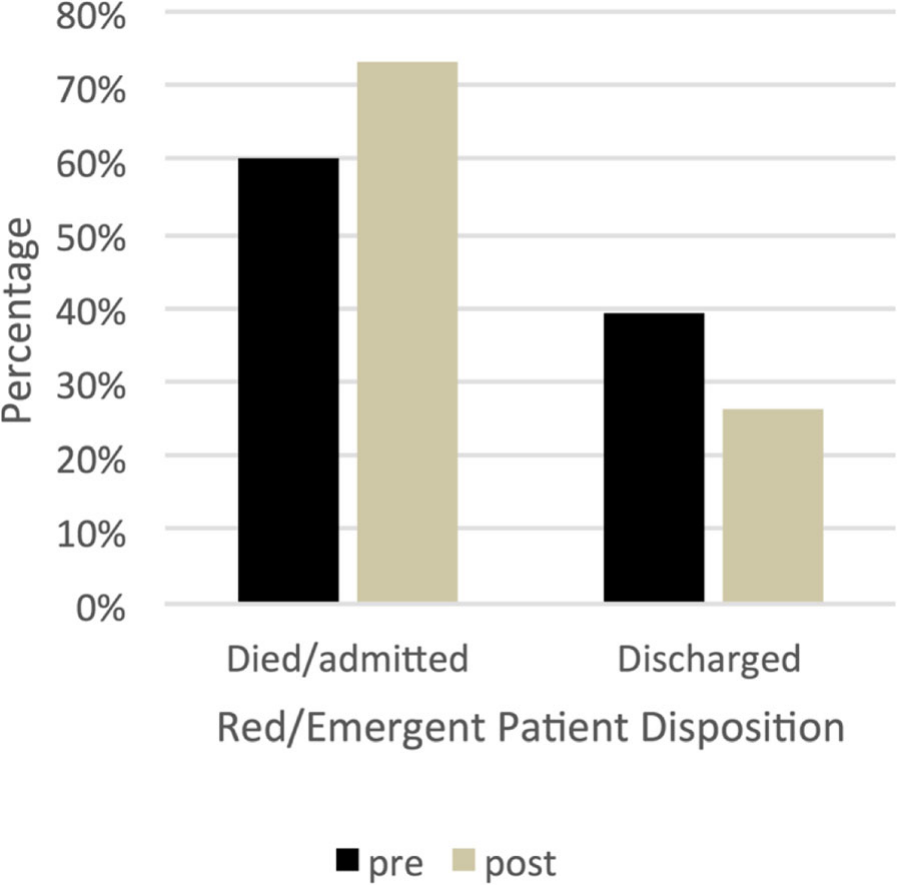
Proportion of triage level “emergent” patients who died or admitted, or were discharged, pre- and post-SATS

For our analysis of overtriage, the disposition of Red patients (pre-SATS) were compared to the combined emergent and very urgent (post-SATS) patients, in line with prior research. However, this definition of overtriage is inherently limited for comparing the prior three-level triage practice to a new four-level system. This challenge was also encountered at the Princess Marina Hospital in Botswana, in their transition to the SATS [32]. We have attempted to better match this comparison by specifically reporting the highest acuity patient categories for pre-SATS (Red) and post-SATS (emergent patients) in Fig. 2.

Finally, we did not assess resource utilization or A&E/in-hospital length of stay in relation to the SATS in this study. The true performance of SATS at KNH may be better understood with tracking additional variables such as wait times, time to provider, time to intervention of clinical conditions, adverse events, and final outcomes of discharged patients [12, 41–43]. These variables, and the extent that triage providers actually followed all aspects of the SATS algorithm, would be useful data to further validate this triage system in our context.

## Conclusion

This project uniquely addresses one of the foundational challenges of acute care in sub-Saharan Africa, as outlined by consensus from the African Federation for Emergency Medicine (AFEM). Our results demonstrate that the South African Triage Scale [44] can be effectively implemented in a tertiary public hospital in the East African setting of Kenya. Implementing the SATS in other public hospitals in the region may provide further standardization of triage in Kenya.

## Additional file

Additional file 1: Supplemental: triage definitions. (DOCX 20 kb)

## Abbreviations

A&Es: Accident and emergency departments
ACS-COT: American College of Surgeons – Committee on Trauma
AFEM: African Federation for Emergency Medicine
EMS: Emergency medical services (pre-hospital)
EMT: Emergency medical technician
ICC: Intraclass coefficient
KNH: Kenyatta National Hospital
LMIC: Low- and middle-income country
QWK: Quadratic weighted kappa
SATS: South African Triage Scale
