# Supplementary material for: Implementation and performance of the South African Triage Scale at Kenyatta National Hospital in Nairobi, Kenya
Source: Int J Emerg Med. 2019 Feb 11;12:5. doi: 10.1186/s12245-019-0221-3 (PMC6371470; doi:10.1186/s12245-019-0221-3)
Supplement: Supplementary file 1 — Supplemental: triage definitions. (DOCX 20 kb) [file 12245_2019_221_MOESM1_ESM.docx]

****Supplemental:** triage definitions

**Under and Overtriage Formula**^34,35^

(1-Sensitivity and 1-Specificity)

- Undertriage % (1-Sensitivity) =

(patients triaged low acuity who were acuity high acuity/all high acuity patients)

Routine (or Green) Admitted + Routine (or Green) Died in A&E

All Admitted + All Died in A&E patients

- Overtriage % = (1-Specificity)

(patients triaged high acuity who were actually low-acuity/all low acuity patients)

Emergent (or Red) + Very Urgent + Urgent (or Yellow) Discharged

All Discharged patients

- Positive Predictive Value (PPV) = True Positive/True Positive + False Positive

(proportion of patients triaged high acuity that were admitted or died in A&E)

Emergent (or Red) + Very Urgent + Urgent (or Yellow) Admitted or Died

Emergent (or Red) + Very Urgent + Urgent (or Yellow) Admitted or Died + Routine Admitted

- Negative Predictive Value (NPV) = True Negative/True Negative + False Negative

(proportion of patients triaged low acuity that were discharged)

Routine Discharged

Routine Discharged + Routine Admitted
